# Uterine Leiomyosarcoma Diagnosed at Caesarean Myomectomy: A Case Report

**DOI:** 10.7759/cureus.96468

**Published:** 2025-11-10

**Authors:** Khalid Alloush, Sara El Hamamy, Richard Peevor, Tabitha Oosterhouse

**Affiliations:** 1 Obstetrics and Gynaecology, Betsi Cadwaladr University Health Board, Bangor, GBR; 2 Gynaecological Oncology, Betsi Cadwaladr University Health Board, Bangor, GBR

**Keywords:** antenatal counselling, caesarean myomectomy, hysterectomy, uterine fibroid, uterine leiomyosarcoma

## Abstract

A 33-year-old patient had a history of a uterine leiomyoma that was first diagnosed incidentally on a dating scan in a previous pregnancy. This was further confirmed on different ultrasound scans after birth, and her main symptom was abnormal uterine bleeding. When she was due for further imaging and hysteroscopy, she was already eight weeks pregnant. Throughout pregnancy, the leiomyoma was growing on serial ultrasound scans, but she was not symptomatic. During her elective caesarean section (CS), a pedunculated leiomyoma was protruding from the posterior wall of the lower uterine segment and was excised and sent for histopathology. This was reported as a uterine leiomyosarcoma (ULMS), and she was referred to gynaecological oncology for management. Following this, she had staging investigations, which showed no metastasis. She proceeded to have a hysterectomy, and the histology confirmed complete treatment.

## Introduction

Sarcomas represent around 3-7% of uterine cancers. Leiomyosarcoma originates from the smooth muscles and is one of the most common sarcomas, representing 1-2% of uterine cancers. Uterine leiomyosarcoma (ULMS) is mainly diagnosed in perimenopausal and postmenopausal people; however, it is still rarely diagnosed in childbearing age. The clinical picture can mimic uterine leiomyoma and include abdominal pain, abnormal uterine bleeding, and/or a pelvic mass [1]. Therefore, the diagnosis of ULMS is challenging; however, suspicion usually arises with rapidly growing leiomyoma, especially in perimenopause or with suspicious features on ultrasound or magnetic resonance imaging (MRI). Definitive diagnosis depends on histopathological examination. Standard treatment of ULMS is hysterectomy and bilateral salpingo-oophorectomy; however, lymphadenectomy can be considered in cases of bulky or suspicious nodes [2].

## Case presentation

A 33-year-old, gravida 3, para 1 patient was booked to the antenatal clinic with a dichorionic diamniotic twin pregnancy and a longstanding history of a uterine leiomyoma. This leiomyoma was first diagnosed incidentally on a dating scan in her first pregnancy, reported as a 25×18×21 mm heterogeneous mass in the myometrium. She had an uncomplicated pregnancy and gave birth by caesarean section (CS) due to a delay in the first stage of labour. A few years later, she started complaining of intermenstrual and heavy menstrual bleeding and had a repeat ultrasound, which revealed a 32×23×31 mm heterogeneous vascular mass, and it was unclear if it was confined to the endometrium or breaching the endometrial/myometrial interface. Following this, an endometrial biopsy was obtained and reported as normal proliferative endometrium with no evidence of hyperplasia, atypia, or neoplasia. She then had an outpatient hysteroscopy, which showed indentation in the uterine cavity but no polyps or leiomyomata. She was counselled regarding the management options, but due to the small size of the leiomyoma, she preferred expectant management and was discharged to primary care.

**Figure 1:**
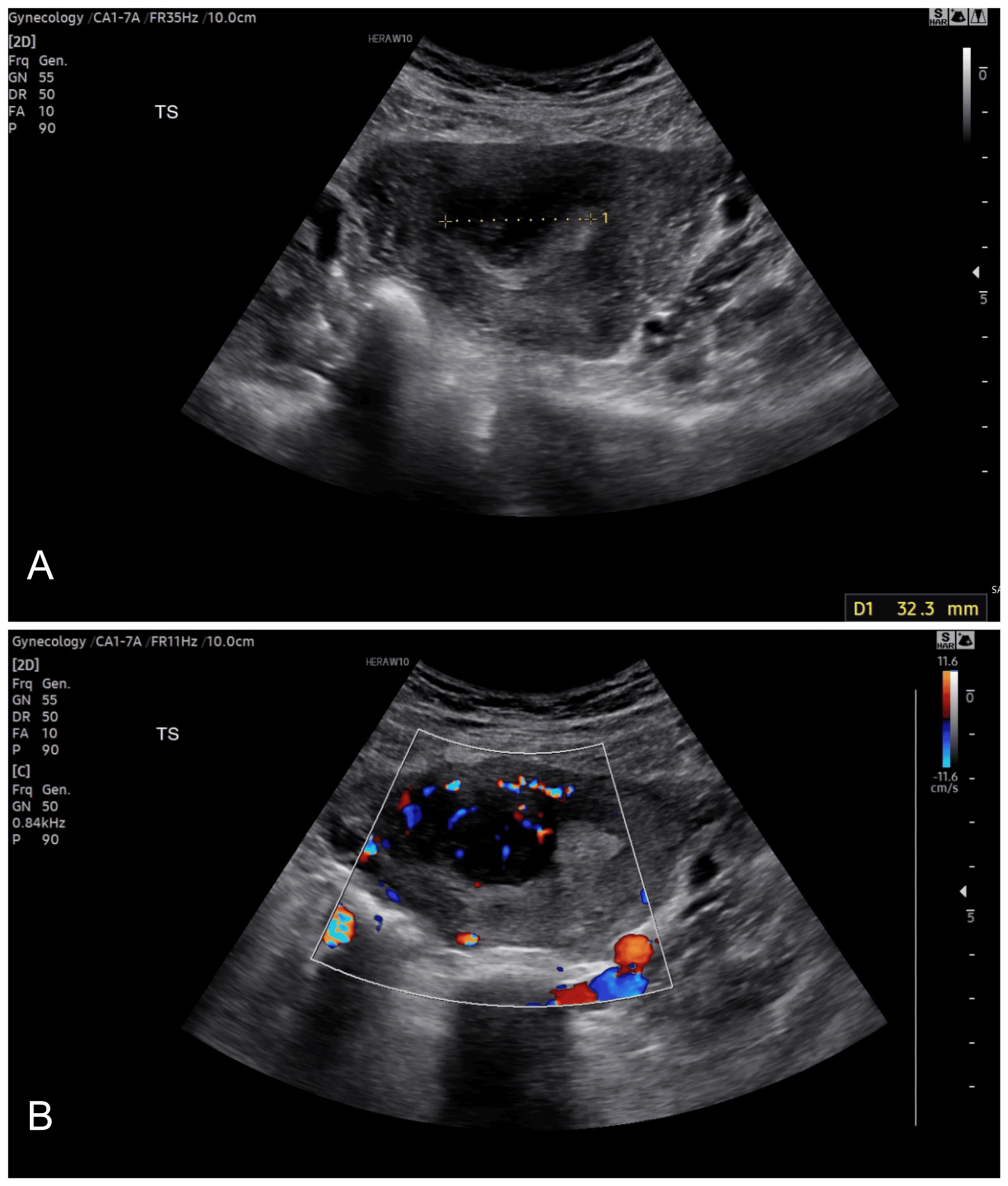
Ultrasound following miscarriage and persistent vaginal bleeding raising suspicion of an abscess showing (A) a uterine heterogeneous mass and (B) high vascularity on Doppler examination

After her second pregnancy unfortunately ended in a miscarriage, she underwent surgical management under general anaesthesia. Following this, she had persistent vaginal bleeding for more than six weeks, and an ultrasound scan showed a 32 mm heterogeneous vascular mass with suspicion of an abscess (Figure 1), which was managed with antibiotics.

**Figure 2:**
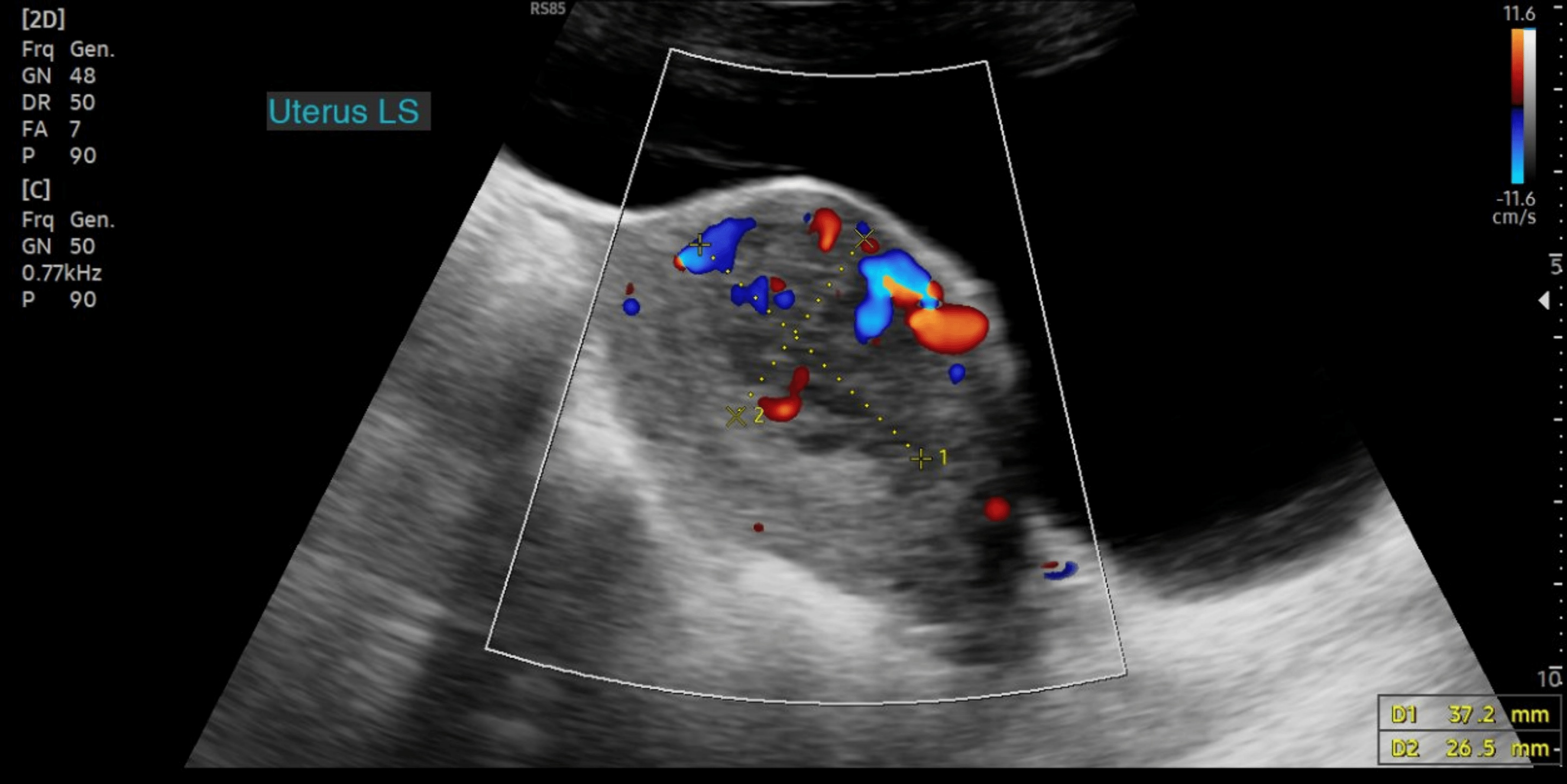
Ultrasound showing heterogenous highly vascular mass at the anterior endometrial/myometrial interface

A year later, when her symptoms of abnormal uterine bleeding persisted, she was referred again to secondary care to discuss her options. A repeat ultrasound showed a heterogeneous, highly vascular mass measuring 37×26 mm situated at the anterior endometrial/myometrial interface and reported as an atypical appearance for a leiomyoma (Figure 2). After reviewing the ultrasound, the plan was for further imaging and possibly repeat hysteroscopy; however, four weeks after this scan, she found she was pregnant.

**Figure 3:**
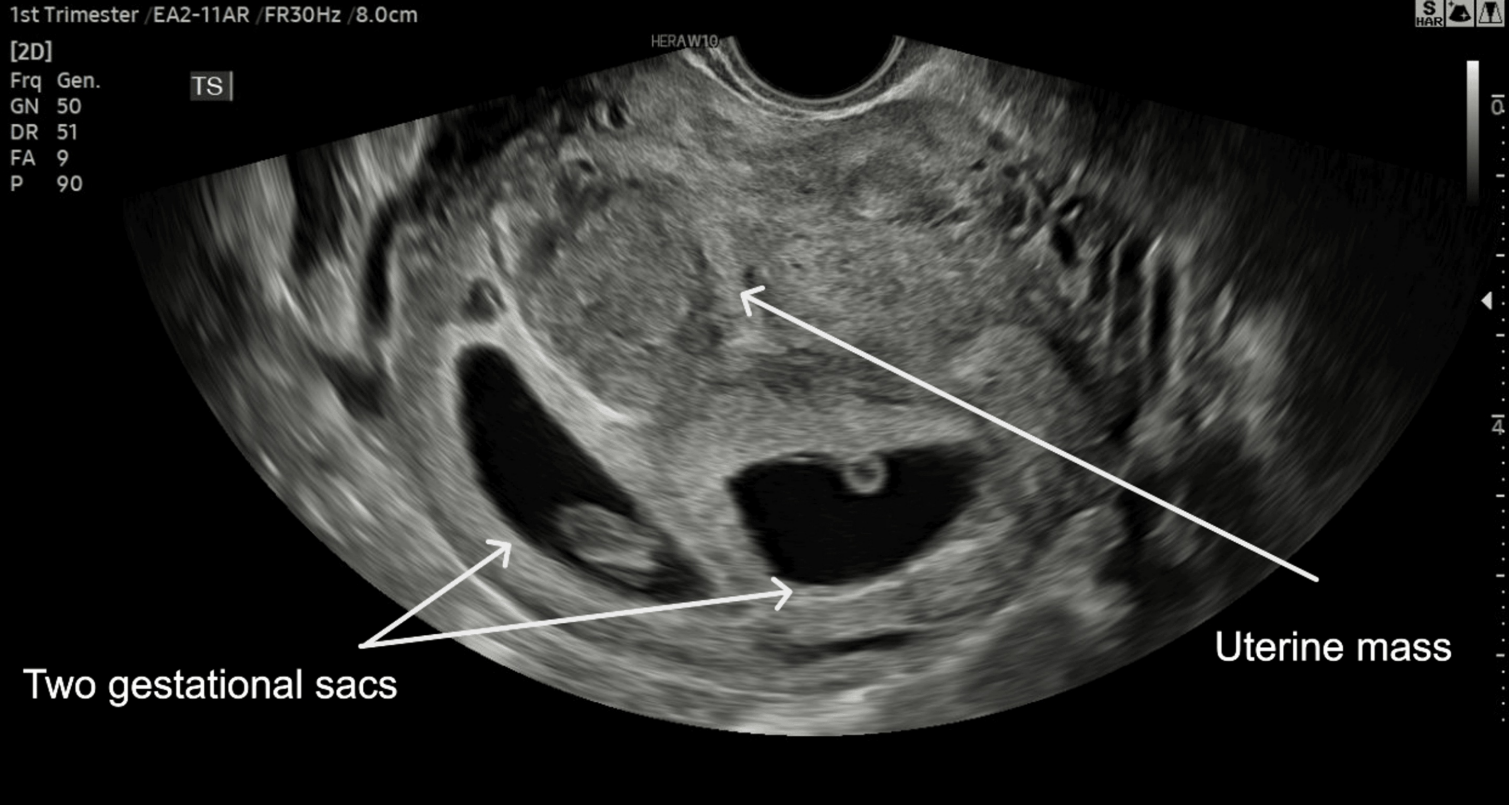
Ultrasound at seven weeks of gestation showing twins and the same uterine lesion

**Figure 4:**
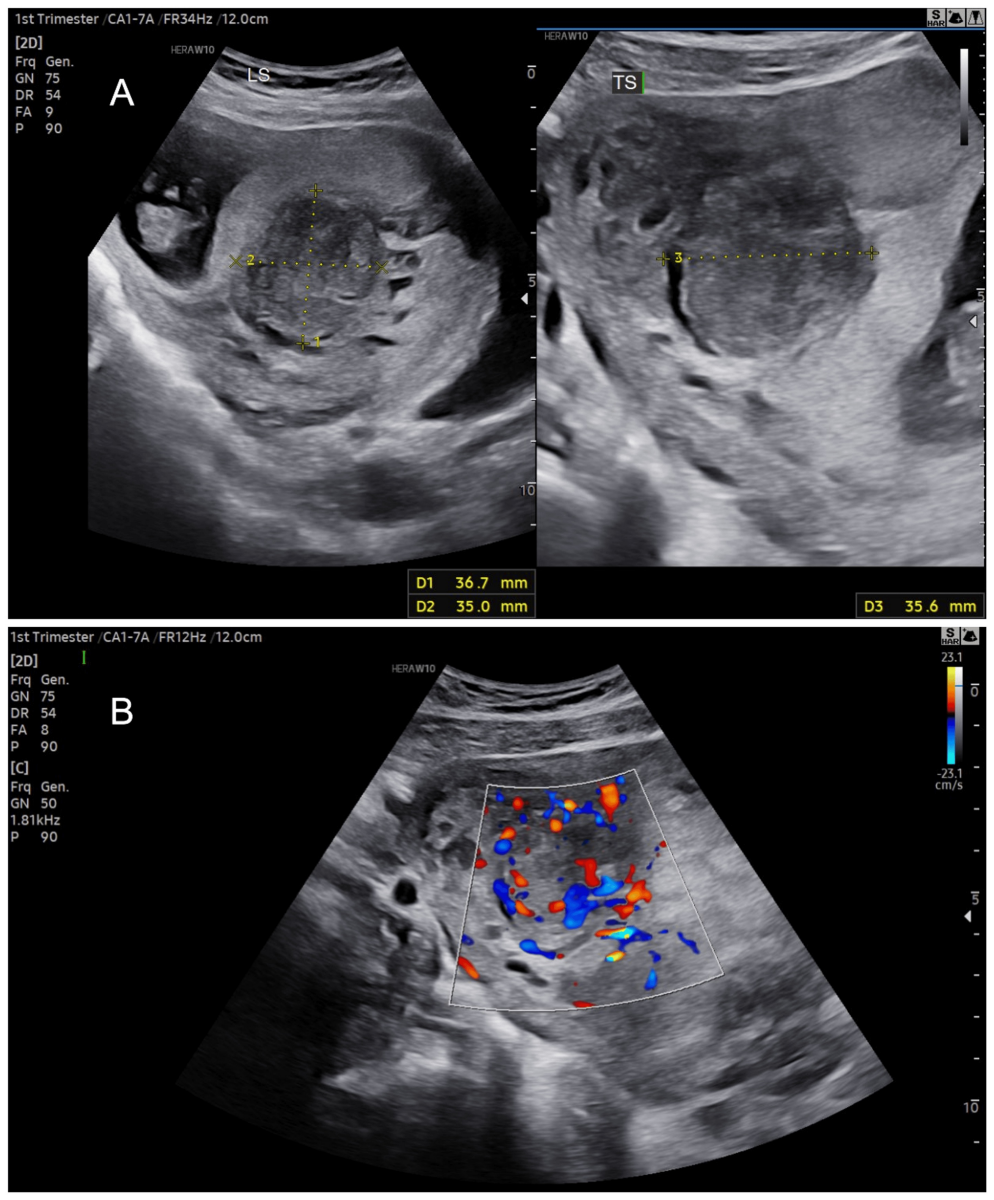
Ultrasound at nine weeks of gestation showing (A) the uterine mass and (B) high vascularity on Doppler examination

Throughout pregnancy, she had serial scans, and the leiomyoma was noted to be growing, but she was asymptomatic (Figures 3-4). She was counselled about the changes in the leiomyoma, and a plan was made for a biopsy or excision if possible, at her CS. At 37 weeks of gestation, she underwent an elective repeat CS. After delivery of the babies and placentae, a pedunculated leiomyoma was found protruding from the posterior wall of the lower uterine segment and was easily accessible. The leiomyoma was excised and sent for histopathology, and bleeding was controlled using haemostatic sutures at its base.

**Figure 5:**
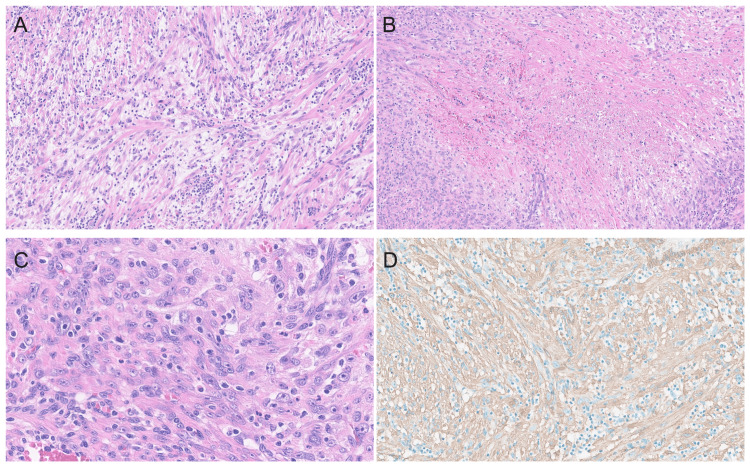
Histopathology of the excised lesion showing (A) pleomorphic spindle cell tumour, (B) areas of necrosis, (C) mitotic figures, and (D) immunohistochemical stain for smooth muscle actin

Following this, the histopathology showed decidualised fibromuscular tissue with intermediate trophoblasts and chorionic villi. In addition, there were pleomorphic spindle cells and numerous mitotic figures, approximately 4 per 10 HPF (Figure 5). Foci of necrosis were present, nuclear atypia was mild to moderate, and there was a diffuse inflammatory infiltrate throughout the neoplastic lesion. Due to the unusual findings, the slides were reviewed and confirmed at a tertiary hospital. The atypical spindle cells were positive for smooth muscle actin, desmin, and caldesmon and weakly positive for PR, CD10, p16, and cyclin D1. The p53 showed a normal, wild-type immunoprofile. This led to the diagnosis of ULMS, and the patient was urgently referred to gynaecological oncology for management.

The patient was counselled about the diagnosis of ULMS, potential risks, and management options. She underwent further investigations, including MRI of the pelvis and computed tomography (CT) of the thorax, abdomen, and pelvis, with no evidence of metastasis, and an International Federation of Gynecology and Obstetrics (FIGO) stage I was the provisional diagnosis. After the multidisciplinary team recommended surgery and the patient was counselled, she underwent a total laparoscopic hysterectomy and salpingectomy with the conservation of ovaries. The specimen was sent for histopathology, and the report confirmed no residual malignancy but showed incidental cervical intraepithelial neoplasia (CIN) 3, clear of all margins and adenomyosis. Therefore, she had a follow-up vaginal vault smear after six months, which was negative.

## Discussion

Although the peak incidence of ULMS is in perimenopause, it has been most often reported between the ages of 35 and 75 and can present in childbearing age. As the symptoms are generally similar to leiomyoma, diagnosis is often incidental after myomectomy or hysterectomy. It can present with abnormal uterine bleeding (56%), palpable pelvic mass (54%), or pelvic pain (22%). The suspicious features include accelerated growth of the leiomyoma, especially in postmenopausal people [1]. It remains difficult to distinguish ULMS from leiomyoma using imaging, but there may be certain features that raise suspicion. Suspicious features on ultrasound include the presence of cystic areas, irregular borders, moderate to rich vascularity, and heterogeneity [3,4]. This should instigate pelvic MRI for the characterisation of the lesion, and key features on MRI include irregular borders, areas of haemorrhage or necrosis, heterogeneous and intermediate to high intensity on T2-weighted images, and low intensity with areas of high intensity on T1-weighted images [4]. Histopathological examination remains the gold standard in differentiating ULMS from leiomyoma. They typically show nuclear atypia, high mitotic activity, and areas of tumour necrosis. Moreover, they stain positive for smooth muscle-specific markers such as actin and desmin [5].

Due to estrogen and progesterone being key hormones in promoting leiomyoma growth, it has been argued that it can increase in size in pregnancy due to the increased levels of hormones. Evidence suggests that around 25% of leiomyomata grow in pregnancy, especially in the first trimester [6]. However, although it is less likely for this change in size to be due to malignancy, it is safer to consider it as a possibility. Antenatal counselling should cover the implications of the leiomyoma, the potential changes, and the possibility of caesarean myomectomy.

Caesarean myomectomy has been a controversial topic with many studies discussing the benefits and risks associated with it. The argued benefits include treatment of leiomyoma and avoiding a possible later surgery for myomectomy. A few articles concluded that caesarean myomectomy can be safe and cost-effective, specifically if the leiomyoma is causing difficulty in the delivery of the fetus or the closure of the uterine incision, of large size (>6 cm) or subserosal [7]. However, the latest Cochrane systematic review still provides uncertainty about its benefits and risks, specifically bleeding, infection, and blood transfusion, as the available evidence is of very low certainty [8].

Looking at the literature, there are 10 articles reporting cases of ULMS diagnosed in pregnancy or puerperium (Table 1) [9-18]. All the patients in these reports underwent abdominal hysterectomy except for one who preferred conservative management after myomectomy to preserve fertility and had no complications [15]. These articles demonstrate the difficulty of distinguishing ULMS from leiomyoma, as the main symptoms were an increase in the size of the leiomyoma and pelvic pain. This again emphasises the value of considering the risk of malignancy and antenatal patient counselling.

**Table 1:** Outcomes in articles reporting ULMS in pregnancy CS: caesarean section; ULMS: uterine leiomyosarcoma

Year published	Presentation in pregnancy	Pregnancy outcome	Specimen	Ovaries	Lymph nodes removed	Metastasis	Adjuvant therapy
This case report	Growing leiomyoma	CS at 37 weeks	CS myomectomy	Preserved	No	No	No
2021 [9]	Growing leiomyoma	CS at 38 weeks	CS myomectomy	Removed	Yes	No	No
2017 [10]	Known leiomyoma	CS hysterectomy at 31 weeks for placenta praevia accreta	CS hysterectomy	Preserved	No	Yes (lung)	Chemotherapy
2008 [11]	Incidental	CS at 34 weeks	CS myomectomy	Removed	Yes	No	Chemotherapy
2005 [12]	5 cases of known leiomyomata	1 vaginal birth at 38 weeks; 4 CS	Postpartum hysterectomy	Preserved	Yes	No data	No data
1999 [13]	Growing leiomyoma	CS at 33 weeks	CS hysterectomy	Removed	No	Yes	No
1994 [14]	Incidental finding after spontaneous pregnancy loss	Spontaneous pregnancy loss at 25 weeks	Evacuation of suspected retained products	Removed	Yes	No	No
1994 [14]	Abdominal pain in pregnancy	Emergency CS at 35 weeks	Postpartum hysterectomy; suspected pelvic abscess and uterine perforation	No data	No	Yes	No
1990 [15]	Incidental	Emergency CS at 33 weeks	CS myomectomy	Preserved	No	No	No
1989 [16]	Abdominal pain; known leiomyoma	Emergency CS at 34 weeks	CS myomectomy	Preserved	No	No	Chemotherapy
1980 [17]	Incidental	Termination of pregnancy in the first trimester	Products of conception	Removed	No	No data	No data
1969 [18]	Severe bleeding in early pregnancy	Emergency surgical management of miscarriage	Products of conception	Removed	No	No data	No data

Management of FIGO stage I ULMS is total hysterectomy with bilateral salpingo-oophorectomy, and while the standard approach is open surgery, a minimally invasive approach can be considered if the integrity of the uterus can be assured. Moreover, in premenopausal people, ovarian preservation can also be considered after counselling the patients and discussing the risks and benefits. Chemotherapy may be discussed with the patient, but there is uncertainty about its benefit; however, radiotherapy is not recommended. The role of chemotherapy and radiotherapy is more significant in more advanced stages and recurrence [2].

Looking back at our patient, she became pregnant after having her scan and could not have further investigations. Had she had ULMS diagnosed before pregnancy, she would have had the same management by hysterectomy. Fortunately, it did not progress during pregnancy, she had two healthy babies, and she did not need to have extra treatment due to a delay in management.

## Conclusions

Leiomyosarcoma is a rare uterine malignancy that arises from the smooth muscle layer of the uterus. It should be suspected in a rapidly growing leiomyoma or persistent abnormal uterine bleeding. Patients with atypical leiomyoma findings on ultrasound should be offered an MRI. It remains difficult to distinguish ULMS from leiomyoma, and histological assessment is the cornerstone of diagnosis.

Caesarean myomectomy can be considered in large, pedunculated, or lower-segment leiomyoma or a leiomyoma with suspicious features. Counselling the patients antenatally about the possible changes in pregnancy and the potential need for myomectomy is recommended.
